# RealNeuralNetworks.jl: An Integrated Julia Package for Skeletonization, Morphological Analysis, and Synaptic Connectivity Analysis of Terabyte-Scale 3D Neural Segmentations

**DOI:** 10.3389/fninf.2022.828169

**Published:** 2022-03-02

**Authors:** Jingpeng Wu, Nicholas Turner, J. Alexander Bae, Ashwin Vishwanathan, H. Sebastian Seung

**Affiliations:** ^1^Princeton Neuroscience Institute, Princeton University, Princeton, NJ, United States; ^2^Department of Computer Science, Princeton University, Princeton, NJ, United States; ^3^Department of Electrical and Computer Engineering, Princeton University, Princeton, NJ, United States

**Keywords:** skeletonization, morphological analysis, clustering, connectomics, Julia language, neuron morphology, neuron connectivity

## Abstract

Benefiting from the rapid development of electron microscopy imaging and deep learning technologies, an increasing number of brain image datasets with segmentation and synapse detection are published. Most of the automated segmentation methods label voxels rather than producing neuron skeletons directly. A further skeletonization step is necessary for quantitative morphological analysis. Currently, several tools are published for skeletonization as well as morphological and synaptic connectivity analysis using different computer languages and environments. Recently the Julia programming language, notable for elegant syntax and high performance, has gained rapid adoption in the scientific computing community. Here, we present a Julia package, called RealNeuralNetworks.jl, for efficient sparse skeletonization, morphological analysis, and synaptic connectivity analysis. Based on a large-scale Zebrafish segmentation dataset, we illustrate the software features by performing distributed skeletonization in Google Cloud, clustering the neurons using the NBLAST algorithm, combining morphological similarity and synaptic connectivity to study their relationship. We demonstrate that RealNeuralNetworks.jl is suitable for use in terabyte-scale electron microscopy image segmentation datasets.

## Introduction

Neural morphology and synaptic connectivity are closely related to brain function. With both nanometer resolution and a large field of view, advanced Electron Microscopes can produce large-scale image stacks (Kornfeld and Denk, 2018; Yin et al., 2020). Image voxels, pixels in a 3D image volume, can be clustered as individual neurons manually (Kasthuri et al., 2015) or automatically using computer vision technologies (Lee et al., 2017, 2019, 2021; Januszewski et al., 2018; Macrina et al., 2021). Benefiting from the rapid development of deep learning (LeCun et al., 2015), the performance of automated segmentation approaches has greatly improved (Beier et al., 2017; Lee et al., 2017, 2019). With additional help from proofreading (Kim et al., 2014; Zhao et al., 2018; Dorkenwald et al., 2020; Hubbard et al., 2020), reconstructed neurons with synaptic connectivity can be used for scientific discovery (Deutsch et al., 2020; Januszewski et al., 2020; Vishwanathan et al., 2020).

Neurons are like trees and their skeletons can be used for morphological analysis. Skeleton or centerline representation is widely used in the morphological analysis (Stepanyants and Chklovskii, 2005; Halavi et al., 2008; Cuntz et al., 2011; Parekh and Ascoli, 2013; Armañanzas and Ascoli, 2015). In contrast to manual tracing and getting a neuron skeleton directly, most existing automated segmentation methods produce voxel labeling and are skeletonized in another step.

Synapses can also be detected automatically (Huang et al., 2018; Turner et al., 2020; Buhmann et al., 2021; Liu and Ji, 2021). Synaptic connectivity analysis can be used to detect motifs or communities. Although several software tools exist for each processing or analysis step, they were normally implemented using different computer languages. There is a lack of a consistent computational environment for the whole analysis pipeline, and users have to switch back and forth between different programming languages and environments.

Traditionally, developers normally use an interpreted language for prototyping, such as Python or MATLAB (MathWorks, Inc., Natick, MA, United States), and then translate the code to a compiled language, such as C or C++, to speed up the computation for large scale deployment. This was called a “two-language problem.” Although some packages, such as Cython and pypy, can be used to help generate lower-level code, there still exist a lot of restrictions. Recently, a programming language with both intuitive syntax and high performance, called Julia (Bezanson et al., 2017), was designed to tackle this problem and has gotten more and more popular in the scientific computing community (Perkel, 2019). Benefiting from this design, prototype code can be compiled just in time and transformed into efficient binary code. As a result, we do not need to rewrite the prototype code using another low-level language, such as C or C++. Motivated by this elegant design, we use Julia to implement some essential analysis steps, including skeletonization, morphological analysis, and connectivity analysis, in two software packages called RealNeuralNetworks.jl and BigArrays.jl.

## Materials

**FIGURE 1:**
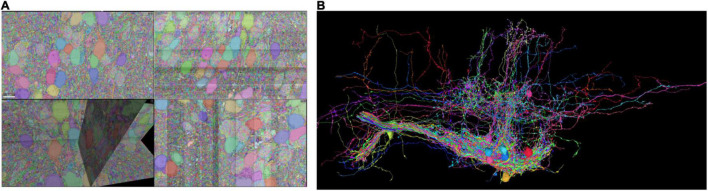
Sparse segmentation after proofreading. **(A)** Some of the neurons are proofread and the fragments are agglomerated as individual neurons. **(B)** Some of the proofread neurons are visualized.

We demonstrate the usage of RealNeuralNetworks.jl by analyzing a dataset with some proofread neurons. The details of this dataset, including sample preparation, imaging, automated segmentation, proofreading, was previously reported (Vishwanathan et al., 2017, 2021). Briefly, a sample (about 250 μm × 120 μm × 80 μm) from a zebrafish larvae brainstem was stained, sectioned, and imaged using a Zeiss Sigma field emitting scanning electron microscope. The image voxel size is 5 nm × 5 nm × 45 nm, and the final image volume size is over four terabytes with a voxel bit-depth of 8 (256 gray levels). Images are aligned and segmented automatically using a convolutional neural network (Lee et al., 2017; Wu et al., 2021). Based on the automated segmentation, about three thousand objects, including neurons or orphan neurites, were proofread using a modified Eyewire system (Kim et al., 2014; Greene et al., 2016; Bae et al., 2018; Vishwanathan et al., 2021). The final plain segmentation was exported to Google Cloud and visualized using Neuroglancer (Maitin-Shepard, 2021; Figure 1).

## Methods and Results

### Data Storage

Segmentation and skeleton data are stored in Google Cloud Storage. The cutout and saving of segmentation chunks are implemented in a standalone Julia package, called BigArrays.jl (see section “Code Availability”). This is similar in functionality to the Python package CloudVolume (Charles et al., 2020; Silversmith et al., 2021b), and the data format is compatible with both packages. The cutout and saving of chunks were implemented on the client, so no intermediate server was needed. Benefiting from the distributed storage system in the cloud, the cutout and saving performance scales linearly with the number of operations. Besides skeletonization, BigArrays.jl was designed for general usage and could be used to handle arrays that are too large to fit in RAM. For example, a potential application is solving the out-of-memory issue in the simulation of quantum computing using tensor networks (Fishman et al., 2020) (Personal Communication).

For skeletonization, we can store the results in several formats. Currently, we support SWC with plain text encoding (Ascoli et al., 2001) which is widely used in most other analysis tools. Additionally, We also created a customized binary representation of SWC and all the numbers are encoded as binary scalar values directly and the loading and saving speed is greatly accelerated. For the synapses, it was detected externally and the result was saved using a language agnostic format “CSV.”

Additionally, the data, including segmentation volume and skeletons, are formatted following Neuroglancer Precomputed. As a result, the data could be visualized directly using Neuroglancer (Maitin-Shepard, 2021) once they are uploaded to the cloud storage without any additional work.

### Distributed Skeletonization of Neurons

To speed up skeletonization, we implemented the hybrid cloud distributed computation architecture in python-based chunkflow (Wu et al., 2021). The object IDs were used to define tasks and all the IDs were ingested to a queue in Amazon Simple Queue Service (SQS) using a Julia package called AWSSDK.jl (2021). The skeletonization of each neuron is independent of each other, so performance scales linearly with the number of nodes allocated.

**FIGURE 2:**
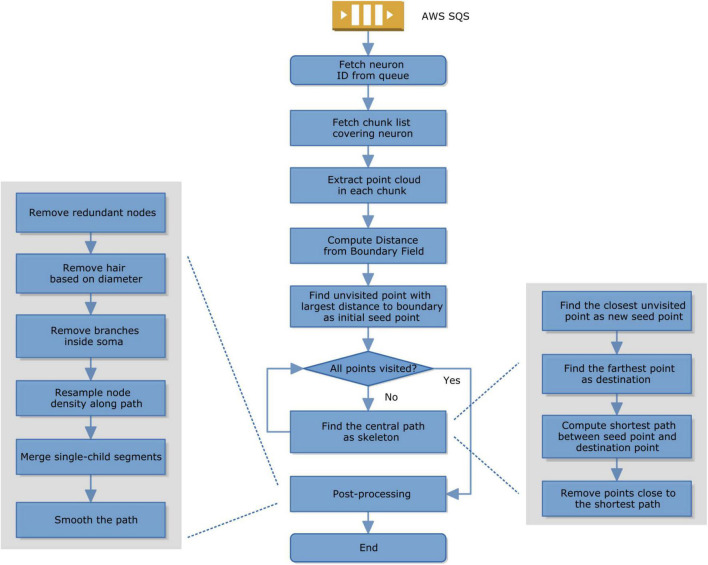
Skeletonization computation in a worker.

Because task management (in SQS) and storage management are both distributed, we can launch workers on any computer with an internet connection and cloud authentication. Each task performs skeletonization for one object, called sparse skeletonization. The computation pipeline on the worker uses a modified TEASAR algorithm (Sato et al., 2000; Bae et al., 2018; Silversmith et al., 2021a). Briefly, the steps are as follows (Figure 2).

1. A worker fetches a task from SQS;
2. It then fetches the segmentation chunk list covering that object or neuron;
3. It extracts the point cloud of that object; It computes the distance from the boundary of the binary mask of that object;
4. It finds a point with the largest distance to the boundary as a seed;
5. If not all the points are visited, find a new central path by computing the shortest path from seed to the furthest unvisited point and then mark all the nearby points as unvisited;
6. If all the points have already been visited, the skeletonization is done and it switches to postprocessing, including removing redundant nodes, removing hair by comparing the diameter and path length, removing branches inside the cell body, resampling the node density to make it more evenly distributed along the path, removing empty branches, smoothing.

Given a sparsely or densely segmented volume, we extract the centerline or skeleton of its neurites one by one using a modified TEASAR algorithm (Sato et al., 2000; Bae et al., 2018; Silversmith et al., 2021a). Given a bit-packed binary volume representing a neuron, the foreground voxels are extracted as a point cloud. The distance from each point to the nearest boundary was computed as a Distance from Boundary Field (DBF). Find the point with the largest DBF as a seed point. Construct an undirected graph with points as nodes and neighboring points are connected with edges. Find the farthest unvisited point as the destination and compute the shortest path as the skeleton using Dijkstra’s algorithm (Dijkstra, 1959). Points around the skeleton are marked as visited and not used in the following computation. Find the unvisited point closest to visited points as the new seed and iterate until all the points are visited. If the segmentation voxel is not continuous, we can look for the nearest terminal node (Supplementary Figure 1) to reconnect within a distance threshold. Note that the binary representation was bit-packed and the memory usage was reduced by 8 fold.

**FIGURE 3:**
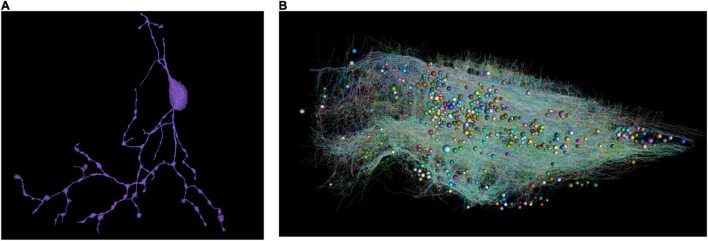
**(A)** Skeletonize of a single neuron. Note that broken parts were reconnected. **(B)** All the skeletons with a random color. The spheres represent cell bodies with varying diameters.

As a result, all proofread neurons are skeletonized (Figure 3). The distributed computation was performed in Google Cloud.

### Morphological Features for Single Neuron Analysis

**FIGURE 4:**
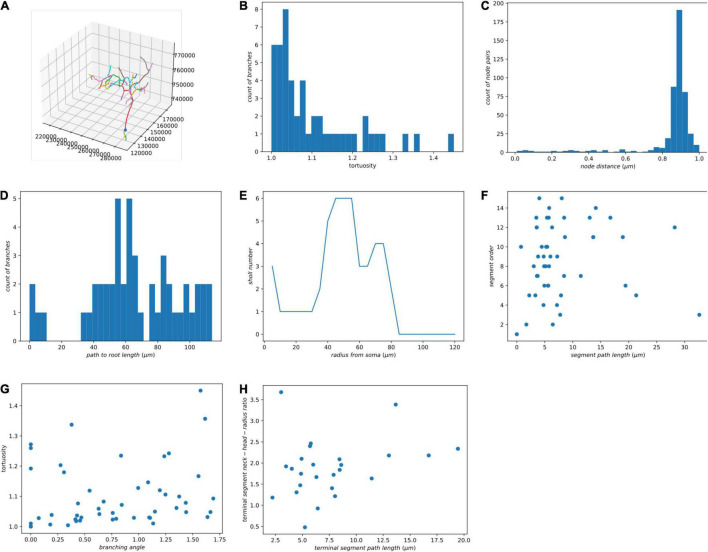
Some morphological features of a single neuron. (A) The morphology of a neuron is visualized in Jupyter Notebook. (B) Histogram of tortuosity of neuron segments. (C) Histogram of neighboring node distance. (D) Histogram of path length to the root node. (E) Sholl analysis. (F) Segment path length versus segment order. (G) Branching angle in radians versus tortuosity of segments. (H) Terminal segment path length versus terminal segment neck-head radius ratio.

We decompose each neuron into segments or single nodes and compute their features. Definitions of node, branching node, root node, terminal node, segment, and the terminal segment are in Supplementary Figure 1. Additionally, an irreducible node corresponds to a soma, branching node, or terminal node. Based on existing literature (Uylings and van Pelt, 2002; Schierwagen, 2008; Schierwagen et al., 2010; Cuntz et al., 2011), we implemented some widely used morphological features for the skeletons and demonstrated the results using our zebrafish dataset (Table 1 and Figure 4). In the spines of mammalian brains, the diameter of the neck is normally much smaller than the head, thus we added a feature to measure the ratio of neck diameter to head (Figure 4H).

**TABLE 1:** Features for single neuron morphology analysis.

Features	Description
Segment order	The order increases from the root node while branching
Segment length	The path length of a single segment
Branching angle	The angle of two segments in a branching point
Tortuosity	The curvature of a segment
Distance to root path length	The minimum path distance from the segment to root node
Average radius	The mean of all the nodes radius in the segment
Radius from soma	For each node, the Euclidean distance from the soma
Terminal segment path length	The path length of each terminal segment
The ratio of neck diameter to head	Could be used to identify spines

### Morphological Features of Many Neurons

For a number of neurons, we would like to encode each neuron using a feature vector, which could be used in neuron type clustering. Based on the literature, we have also implemented several widely used features (Table 2) and applied them to our zebrafish dataset (Supplementary Figure 2; Uylings and van Pelt, 2002; Schierwagen, 2008; Schierwagen et al., 2010; Cuntz et al., 2011; Wanner et al., 2016).

**TABLE 2:** Features of a single neuron.

Features	Description
Distance from soma to the center of skeleton mass	A metric to measure symmetricity centered by soma
Total path length	The physical length of all the skeleton paths
The number of branching points	
Median segment length	The median segment length of all the segments starts and ends at irreducible nodes
3D Sholl Analysis (Sholl, 1953)	Count the intersections to spheres centered on the root node
Average branching radian	The mean of the branching angles
Average tortuosity	The average value of the ratio of the path length to the Euclidean distance between irreducible nodes
Asymmetry	The distance of the soma node to the arbor center of mass
Typical radius	The Euclidian distance of the dendritic arbor points to the center of mass
Fractal dimension	Measures similarity across scales
Root node radius	The radius of the root node which is normally the soma
Total dendrite path length	If the dendrite segments are classified
Longest segment path length	
Convex hull volume	
Surface area	
Post-synapse number	Number of postsynaptic sites
Pre-synapse number	Number of presynaptic sites

### Morphological Clustering Using NBLAST

**FIGURE 5:**
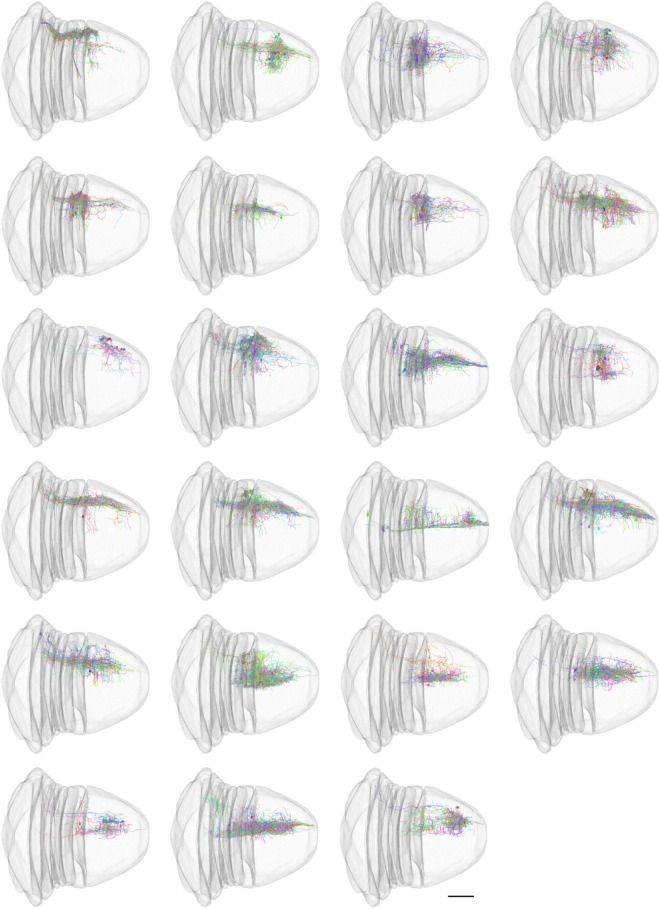
NBLAST classification of neurons. The scale bar in the last image is 100 μm.

Most of traditional morphological features do not measure the spatial distribution of neurons. An automatic neuron type classification method, called NBLAST (Costa et al., 2016), measures the spatial distribution and is getting popular. The original method was implemented in R and C++. In order to incorporate this method in our analysis ecosystem, we implemented this algorithm from scratch using Julia. We performed hierarchical clustering (Supplementary Figure 3) using Clustering.jl (Stukalov and Lin, 2021) and classified the neurons into 23 types based on the NBLAST similarity scores (Figure 5). The visualization was created using Neuroglancer.

### Synaptic Connectivity Combined With Morphology

**FIGURE 6:**
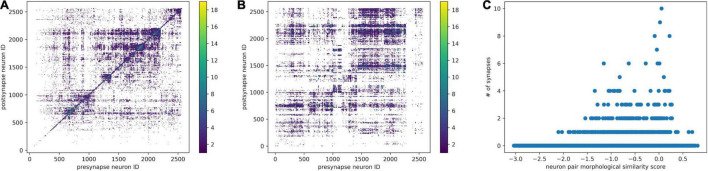
Combine morphological NBLAST clustering and synaptic connectivity. **(A)** The synaptic connectivity matrix was reordered by hierarchical clustering based on connectivity distance. **(B)** The synaptic connectivity matrix was reordered according to hierarchical clustering based on the NBLAST score. The synapse number is encoded in the point diameter and color. **(C)** For each neuron pair, the relationship between NBLAST morphological similarity and number of synapses.

After neuron segmentation and synapse detection were done externally, we can construct a graph of the neural network. Within the graph, the neurons are nodes and the synapses are edges. We use the synapse number as a connectedness metric for neurons. The more synapses connecting two neurons, the closer they are. Based on the distance matrix, we can perform hierarchical clustering, reorder the connectivity matrix, and identify some communities (Figure 6A).

Once we have the skeleton morphological features and synaptic connectivity, we can combine them. We can order the neurons in the connectivity matrix using NBLAST hierarchical clustering. As a result (Figure 6B), there are some morphologically similar neurons highly connected with each other. Morphologically similar neurons tend to have stronger synaptic connections as well (Figure 6C), which is consistent with previous findings in the mouse visual cortex (Lee et al., 2016).

## Discussion

RealNeuralNetworks.jl was built to process voxel segmentation datasets from Serial Section Electron Microscopy images. Some components, such as skeletonization and morphological analysis, can be reused for sparsely labeled neurons in Light Microscopy images.

### Comparison With Related Tools

Most existing tools are specifically designed for one or two analysis steps, rather than providing a one-stop solution and a consistent computational environment. Compared with some related software, RealNeuralNetworks.jl has a more complete toolset for the analysis (Table 3).

**TABLE 3:** Comparison of software tools.

Tool/Feature	References	Language	Skeletonization	Morphological features	NBLAST similarity	Synaptic connectivity
L-Measure	Scorcioni et al., 2008	Java		✓		
NBLAST	Costa et al., 2016	R, C++			✓	
NeuroM	Palacios et al., 2021	Python		✓		
NeuTu	Zhao and Plaza, 2014	C++	✓			
TREES toolbox	Cuntz et al., 2010	MATLAB		✓		
Vaa3D	Peng et al., 2014	C++	✓	✓		
CBLAST	Januszewski et al., 2020	Python, R, C++			✓*	✓
3D BrainCV	Wu et al., 2014	MATLAB	✓	✓		
Kimimaro	Silversmith et al., 2021a	Python, C++	✓			
RealNeuralNetworks.jl		Julia	✓	✓	✓	✓

*CBLAST uses NBLAST for similarity measure.

NeuTu (Zhao et al., 2018) was built mainly for proofreading neuron reconstruction from Electron Microscopy images. Besides that, it can also measure neuron shape similarity and perform clustering of neuron types (Zhao and Plaza, 2014). The measurement is built upon arbor density maps which is much more computationally heavy than skeleton-based NBLAST (Costa et al., 2016). Although the sparse skeletonization of NeuTu was also built upon the TEASAR algorithm (Sato et al., 2000), the geodesic distance between neighboring voxels is measured using the image intensity rather than distance map in our implementation. Thus, the skeleton accuracy is correlated with image quality.

Currently, RealNeuralNetworks.jl only has some widely used morphological features and is not as complete as L-Measure (Scorcioni et al., 2008) and TREES toolbox (Cuntz et al., 2010). Vaa3D (Peng et al., 2010, 2014) was built for light microscopy image processing, especially neuron tracing, and has a much richer set of tracing algorithms.

Kimimaro (Silversmith et al., 2021a) was built for dense skeletonization rather than sparse skeletonization. Currently, it does not have a bit-packed binary representation of segmentation volume and requires much more memory for sparse usage.

### Why Julia

Julia is a modern language with nice features for both scientific computing and general programming (Bezanson et al., 2017; Perkel, 2019). It performs just-in-time compilation for the code, so performance can be comparable with C/C++. In addition, it has an intuitive syntax and an interactive programming interface like MATLAB (MathWorks, Inc., Natick, MA, United States), which is useful for prototyping and experiments. It is open-source with a permissive license, so it is much easier to deploy in the cloud compared with commercial languages requiring a license, such as MATLAB (MathWorks, Inc., Natick, MA, United States). Julia can be used interactively in Jupyter Notebooks (The “Ju” is from the name of Julia) (Perkel, 2019). Julia is increasingly popular in the scientific computing community. It has been downloaded over 25 million times and over 5000 packages are registered.

For most of the interpretable languages, such as Python and MATLAB, manipulating single elements in one or nesting loop is normally tens or hundreds of times slower than low-level languages, such as C and C++. For good performance, programmers are limited to using “vectorized” operations which were actually implemented in lower-level languages. In our applications, we perform a lot of voxel manipulations that are hard to express in “vectorized” operations. Benefiting from the just-in-time compilation, all of such operations can be implemented directly in Julia with good performance.

For the computation in local cluster or supercomputer, Julia was designed for distributed computing at the beginning and has gained a dramatic rise in the high-performance computing community. Our packages are expected to be adaptable in a local cluster.

### Limitations

The skeletonization module was designed for sparse skeletonization rather than dense skeletonization. For sparse skeletonization, we can skeletonize some neurons of interest while the proofreading is ongoing. It would be too computationally expensive to iterate over the neurons individually in a terabyte-scale or petabyte-scale image volume. For dense skeletonization, Kimimaro is a better alternative (Silversmith et al., 2021a).

Currently, RealNeuralNetworks.jl only has limited support for visualization, such as functions for skeleton visualization. For more complicated plots, users must build their own scripts or Jupyter Notebooks based on other Julia visualization packages. Compared with the TREES toolbox (Cuntz et al., 2010, 2011), RealNeuralNetworks.jl does not have an interactive skeleton editing interface. Compared with L-Measure, there are some missing morphological features in RealNeuralNetworks.jl.

Julia is a young language with rapid development and adoption in the scientific computing community. However, many of the packages are still evolving and are not yet stable.

## Conclusion

In summary, we present a Julia-based tool, called RealNeuralNetworks.jl, for sparse skeletonization, morphological analysis, and synaptic connectivity analysis. We provide an integrated computational environment for the analysis pipeline. We have demonstrated the utility of this package by processing a Zebrafish segmentation dataset. We hope that it could be useful for other connectomics projects in the future.

## Code Availability

The code is open-sourced in GitHub: https://github.com/seung-lab/RealNeuralNetworks.jl. The BigArrays.jl is available in GitHub as well: https://github.com/seung-lab/BigArrays.jl. The Jupyter Notebooks are available in GitHub: https://github.com/jingpengw/realneuralnetworks-notebook.

## Data Availability Statement

The original contributions presented in the study are included in the article/Supplementary Material, further inquiries can be directed to the corresponding author.

## Author Contributions

JW implemented the software, performed the experiments, and wrote the manuscript. NT translated the MATLAB skeletonization code to Julia. JB improved the TEASAR algorithm and implemented it in MATLAB. AV contributed to sample preparation, imaging, and management of proofreading. HS designed and conceptualized the study. All authors contributed to the article and approved the submitted version.

## Conflict of Interest

HS has financial interests in Zetta AI LLC. This study received assistance from Google, Amazon, and Intel. These companies were not involved in the study design, collection, analysis, interpretation of data, the writing of this article, or the decision to submit it for publication. The remaining authors declare that the research was conducted in the absence of any commercial or financial relationships that could be construed as a potential conflict of interest.

## Publisher’s Note

All claims expressed in this article are solely those of the authors and do not necessarily represent those of their affiliated organizations, or those of the publisher, the editors and the reviewers. Any product that may be evaluated in this article, or claim that may be made by its manufacturer, is not guaranteed or endorsed by the publisher.

## Supplementary Material

Supplementary Figure 1 Nomenclature of neuron skeleton parts.

Supplementary Figure 2 Distribution of morphological features.

Supplementary Figure 3 Hierarchical clustering using the NBLAST score.
