# Supplementary material for: RealNeuralNetworks.jl: An Integrated Julia Package for Skeletonization, Morphological Analysis, and Synaptic Connectivity Analysis of Terabyte-Scale 3D Neural Segmentations
Source: Front Neuroinform. 2022 Mar 2;16:828169. doi: 10.3389/fninf.2022.828169 (PMC8924549; doi:10.3389/fninf.2022.828169)
Supplement: Supplementary Figure 1 — Nomenclature of neuron skeleton parts. [file Data_Sheet_1.pdf]

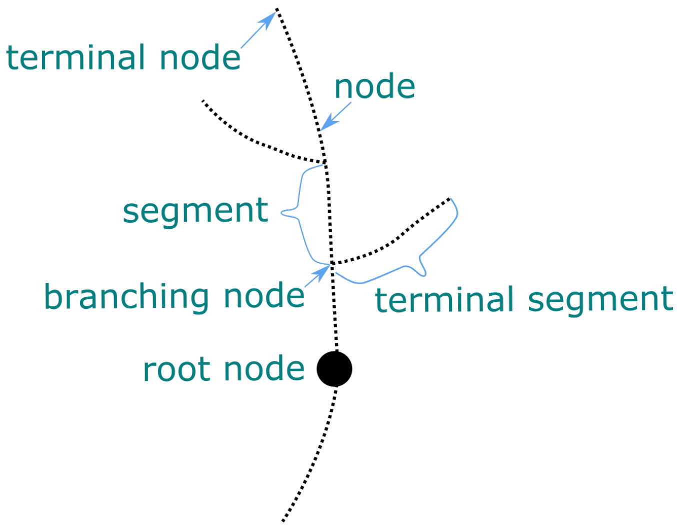

Supplementary Figure 1. Nomenclature of neuron skeleton parts.

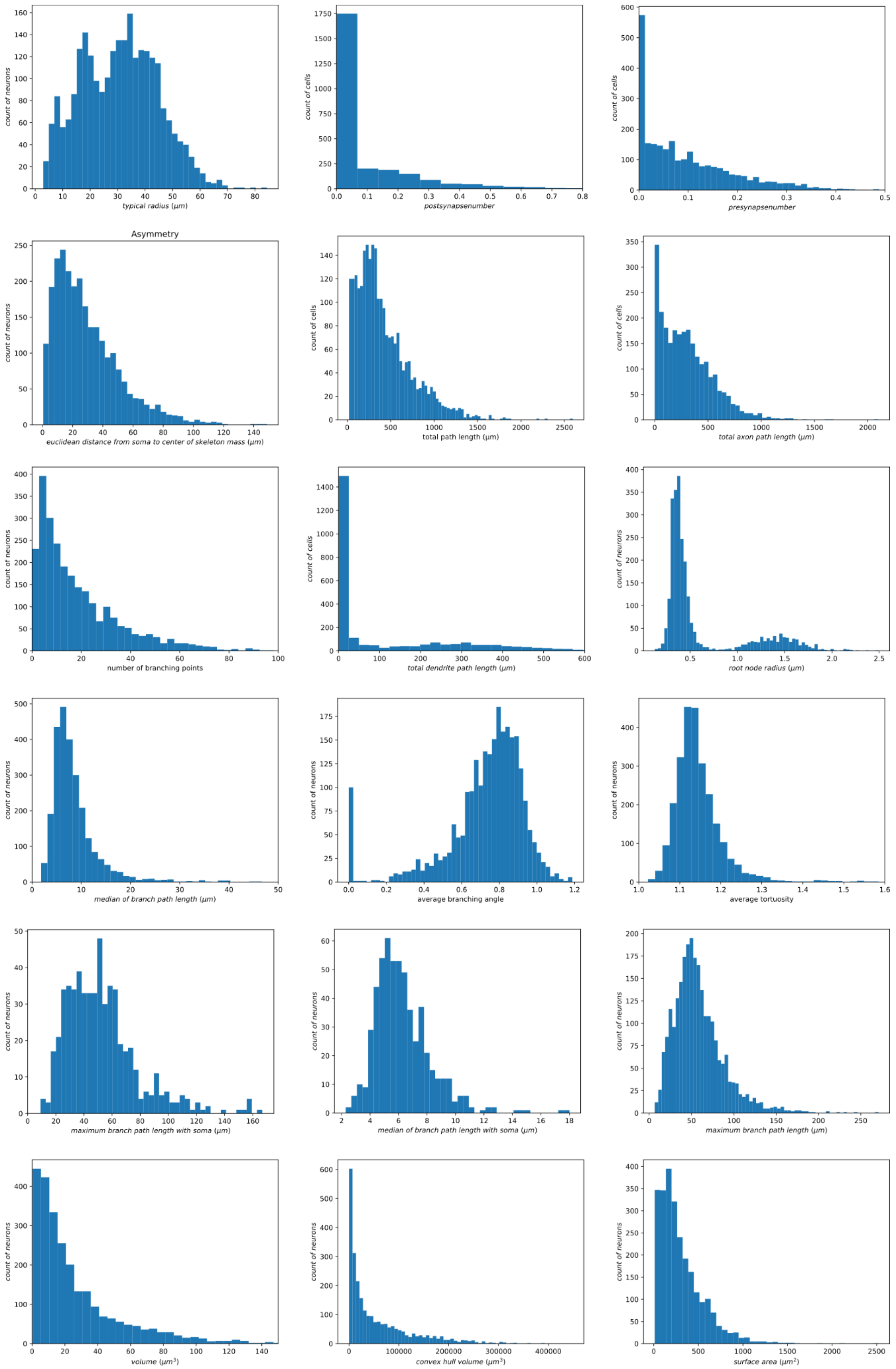

Supplementary Figure 2. Distribution of morphological features.

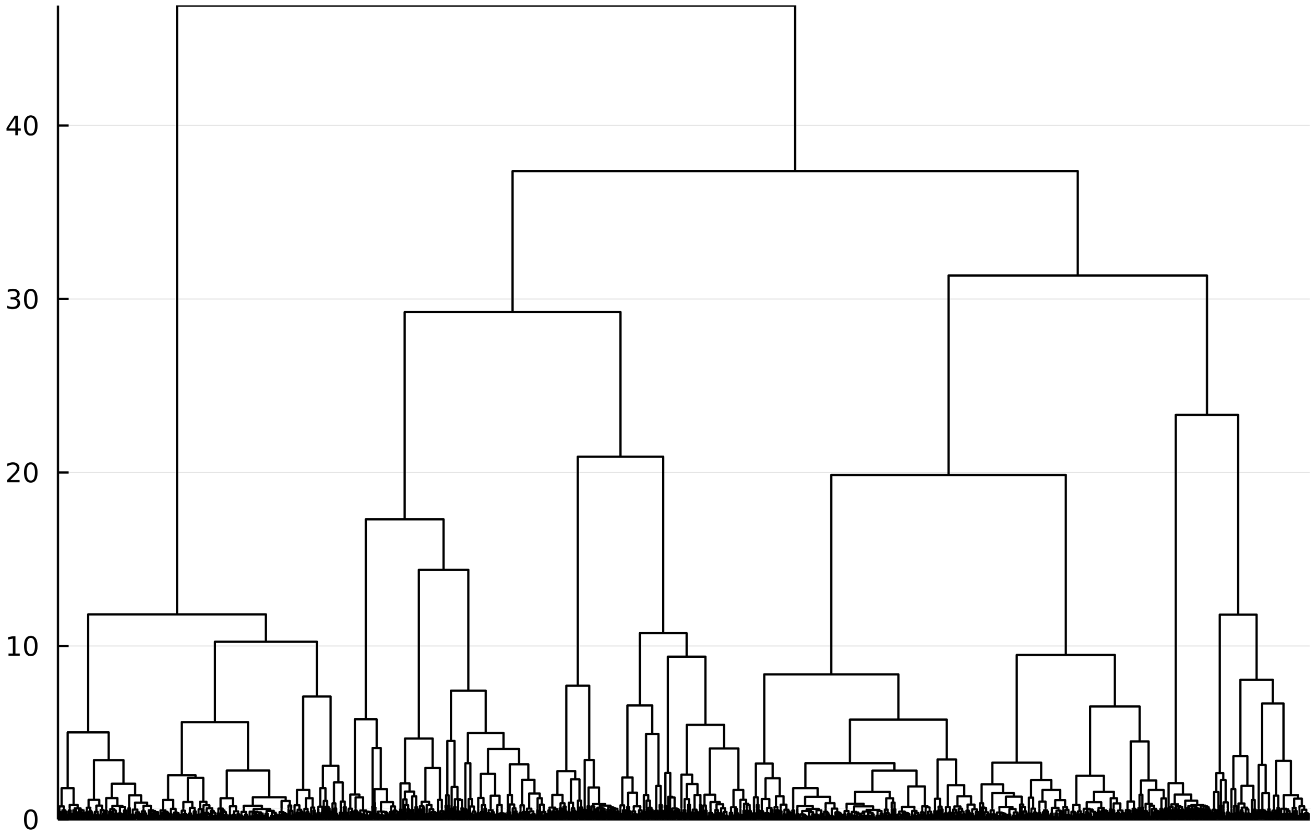

Supplementary Figure 3. Hierarchical clustering using the NBLAST score.
